# A reverse-engineering approach to dissect post-translational modulators of transcription factor’s activity from transcriptional data

**DOI:** 10.1186/s12859-015-0700-3

**Published:** 2015-09-03

**Authors:** Gennaro Gambardella, Ivana Peluso, Sandro Montefusco, Mukesh Bansal, Diego L. Medina, Neil Lawrence, Diego di Bernardo

**Affiliations:** 1 The Telethon Institute of Genetics and Medicine, Naples, Italy; 2 Present Address: Department of Cancer Studies, King’s College London, NHH, London, UK; 3 Columbia Initiative in Systems Biology and Center for Computational Biology and Bioinformatics, Columbia University, New York, NY USA; 4 Department of Computer Science, University of Sheffield, Sheffield, UK

## Abstract

**Background:**

Transcription factors (TFs) act downstream of the major signalling pathways functioning as master regulators of cell fate. Their activity is tightly regulated at the transcriptional, post-transcriptional and post-translational level. Proteins modifying TF activity are not easily identified by experimental high-throughput methods.

**Results:**

We developed a computational strategy, called Differential Multi-Information (DMI), to infer post-translational modulators of a transcription factor from a compendium of gene expression profiles (GEPs). DMI is built on the hypothesis that the modulator of a TF (i.e. kinase/phosphatases), when expressed in the cell, will cause the TF target genes to be co-expressed. On the contrary, when the modulator is not expressed, the TF will be inactive resulting in a loss of co-regulation across its target genes. DMI detects the occurrence of changes in target gene co-regulation for each candidate modulator, using a measure called *Multi*-*Information*. We validated the DMI approach on a compendium of 5,372 GEPs showing its predictive ability in correctly identifying kinases regulating the activity of 14 different transcription factors.

**Conclusions:**

DMI can be used in combination with experimental approaches as high-throughput screening to efficiently improve both pathway and target discovery. An on-line web-tool enabling the user to use DMI to identify post-transcriptional modulators of a transcription factor of interest che be found at http://dmi.tigem.it.

**Electronic supplementary material:**

The online version of this article (doi:10.1186/s12859-015-0700-3) contains supplementary material, which is available to authorized users.

## Background

Modulation of transcriptional regulation in a cell can be exerted at many different levels, including transcription factor (TF) activation/deactivation by post-translation modifications (PTMs). PTMs involve amino-acid residues in a protein that are covalently modified “on the fly”. Through this mechanism, a cell is able to tightly regulate protein function such as its activity, localisation and interaction with other molecules. Capturing this kind of regulatory interactions using only transcriptional data, such as gene expression profiles (GEPs), is considered challenging since GEPs are further downstream of the PTM event and only indirectly linked to it.

Post-translational modulations act as a trigger for many signalling network and thus their alterations are found in many pathologies. Hence, many efforts have been made in the reconstruction of phosphorylation networks from experimental data [1]. These studies have then led to the development of new computational methods to predict the substrate specificities of protein kinases [1–7]. Initially, computational approaches relied on protein sequences in order to identify the consensus motif recognized by the active site of kinase catalytic domain [2–4]. However, such motifs often lack sufficient information to uniquely identify their physiological substrates.

Recently, more sophisticated algorithms have been proposed: Linding et al. [6] developed a analysis pipeline (NetworKIN) to assign experimentally validated phosphorylation sites to specific kinases by combining consensus information from sequence motifs with protein interaction networks. NetworKIN is based on the availability of experimental biochemical data, thus limiting the general applicability of this approach; Wang et al. [5] proposed a reverse-engineering method based on an information-theoretic approach to infer new post-translational modulators of the MYC transcription factor from gene expression profiles. The authors exploited changes in the transcriptional level of a kinase/phosphatase across a set of GEPs to infer the post-translational activation of MYC. Specifically, they developed a computational method (MINDy) based on the estimation of pair-wise Conditional Mutual Information between a TF and its target genes. MINDy detects whether changes in the expression level of a kinase affect the co-expression between a TF and one of its target genes. This method requires the TF and its target gene(s) to be co-expressed across a set of GEPs, at least when the TF is active. Some TFs, however, are not co-expressed with their target genes, thus limiting MINDy applicability.

**Fig. 1:**
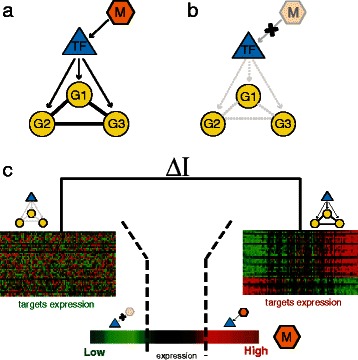
The Differential Multi-Information method. **a** Hypothetical scenario in which a putative Transcription Factor (TF) is activated by phosphorylation or de-phosphorylation through a Modulator (M). G1, G2 and G3 are three downstream targets of the TF. In presence of the Modulator (M) the downstream targets (G1, G2 and G3) become co-regulated through the active Transcription Factor (TF). **b** In absence of the Modulator (M) the downstream targets (G1, G2 and G3) are not co-regulated since the Transcription Factor (TF) is not active. **c** For each iteration of the DMI method a candidate modulator M is tested. First the GEPs are sorted according to the expression level of the modulator M and the GEPs subdivided in three (or more) subsets. The Differential Multi-Information (∆I) of the targets is computed always between the two subset where M expression is either “High” or “Low” by estimating the Renyi Multi-Information and taking its difference

Here, we developed and applied a new reverse-engineering strategy called Differential Multi-Information (DMI or ∆**I** method) to infer post-translational modulators of a TF of interest. Our working hypothesis is the scenario depicted in Fig. 1a–b, in which a modulator (i.e. kinase/phosphatases) when expressed activates the TF. The TF, in turn, will induce concurrent expression changes in its target genes, hence these genes will be co-expressed among themselves (Fig. 1a). On the contrary, when the modulator is not expressed (or not functional), the TF will be inactive and thus not able to regulate its target genes; this will result in a loss of co-expression among target genes (Fig. 1b). DMI requires in input a subset of the TF’s target genes and returns as output a ranked list of predicted modulators. Crucially, DMI does not take into account the TF expression levels, nor it requires the TF to be co-expressed with its target genes.

We applied DMI to an experimental dataset consisting of 5,372 GEPs [8] to identify kinases regulating 14 different transcription factors for which we were able to collect *bona-fide* transcriptional targets.

Our results demonstrate that DMI is able to detect post-translation modulators of TFs from GEPs, thus making it an ideal tool for both basic research and drug discovery.

## Results

### DMI: a Differential Multi-Information approach to the identification of post-translational modula-tors

We developed an algorithm (DMI) to identify post-translational modulators (i.e. kinases or phosphatases) of a Transcription Factor (TF) of interest from gene expression profiles (GEPs). DMI works by detecting changes in the *co-regulation* of the TF’s target genes in the presence or absence of the modulator (Fig. 1a–b). To this end, we computed the Rényi Multi-Information measure (*I*) to estimate the target genes’ co-regulation (*G* ^1^ … *G* ^*d*^) [9] (Material and Methods). Multi-information is a multi-dimensional extension of pair-wise Mutual Information, which quantifies the extent of statistical dependency across a set of *d* variables. A null value of multi-information implies that the *d* variables are statistically independent, whereas positive values correspond to increasing degrees of dependency, i.e. co-regulation.

In order to compute changes in the Rényi Multi-Information *I* of a set of TF’s target genes *G* ^1^ … *G* ^*d*^ in the presence or absence of a modulator M across a set of GEPs, we followed the procedure depicted in Fig. 1c: we first sorted GEPs according to the modulator M’s expression; we then subdivided GEPs into three subsets each containing the same number of profiles. In the first subset (“Low”), the expression level of the modulator M will be lower than in the second subset (“Medium”), which in turn will be lower than in the third subset (“High”). Finally, we computed the Difference in Multi-Information (∆*I*) between the High and Low subsets (Fig. 1c). ∆*I* quantifies how much the modulator M is able to influence the co-regulation of the TF’s target genes. Positive values of ∆*I* imply that when the kinase is present, the TF’s target genes are co-regulated and hence the kinase is able to activate the TF. On the contrary, negative values of ∆***I*** indicate that the kinase is a negative modulator of TF activity. Since M is not known a-priori, ∆***I*** is computed for each modulator M to be tested. The modulators are then ranked by ∆***I*** and by *p*-value, computed using a permutation test, as detailed in the Methods section. The full pipeline of the method is summarised in Additional file 1: Figure S1.

### Validation of DMI “in silico”

We generated two datasets (D1 and D2) consisting of 100 simulated GEPs each. In one half of the GEPs, the TF target genes were co-expressed; in the other half they were assumed to be independent (Material and Methods).

Dataset D1 consists of 60 genes: 10 genes were assumed to be the known targets of the TF; 50 genes were assumed to be the potential modulators M of the TF, but only 20 of them were the effective modulators. In addition, we assumed that 10 of the remaining 30 potential modulators were indeed unknown targets of the TF and hence co-regulated with the TF’s target genes, thus making it harder for the methods to distinguish them from the effective modulators.

Dataset D2 consists of 760 genes. As for dataset D1, only 10 genes were assumed to be the known targets of the TF, whereas the remaining 750 genes were assumed to be potential modulators M of the TF, with only 50 of them being the effective modulators (Material and Methods).

The output of DMI is a list of all the possible modulators (50 in D1 and 750 in D2) ranked according to their differential multi-information, and associated to a *p*-value.

In order to estimate the performance of DMI, we computed the percentage of correct predictions at each position in the rank (also known as Positive Predictive Value—PPV) as PPV = TP/(TP + FP), where TP are the true positives and FP are the false positives. We also computed the fraction of the real modulators discovered at each position in the rank, (also known as Sensitivity) equal to TP/(TP + FN), where FN are the false negatives. A perfect performance would be a constant value of PPV equal to 1.

The results for the first dataset D1 are shown Additional file 1: Figure S2, as a PPV-Sensitivity curve, where the method achieves a perfect performance, i.e. PPV = 1 (Material and Methods).

The results for the dataset D2 are instead reported Additional file 1: Figure S3A, when using either two or three subsets when subdividing the GEPs according to the modulator expression level. Also in this case, the DMI method achieves the best performance ranking the 50 modulators in the top 50 positions.

In order to simulate a more “biologically realistic” scenario and to make it harder for the method to distinguish the modulators present in the dataset, we also generated 4 additional datasets with the same parameters as in D2 but with “noisy bins”. Specifically, in these 4 datasets the number of GEPs in which the TF’s targets are dependent, is either 30, 40, 60 or 70 out of 100 GEPs. Hence, for example, consider the dataset where the targets are dependent in 70 out of 100 GEPs. In this case, when the dataset is discretized in 2 bins with equal number of samples according to the expression of the modulator, the first bin (i.e. low expression of the modulator) should contain only GEPs in which the TF target genes are not co-expressed. However, since this bin will contain 50 GEPs, only in 30 out 50 GEPs the targets will not be co-expressed, thus adding “noise” to the bin.

The PPV-sensitivity curves for these 4 dataset are reported in Additional file 1: Figure S3B-E respectively. In all of the cases tested, DMI performed significantly better than random.

Finally, we also compared Multi-Information measure against other two method used to estimate the dependency among multidimensional variables. As reported in the section “Additional analysis” of supplementary data Multi-Information performed better than those based on pair-wise measures.

### Validation of DMI in human gene expression profiles to identify modulators of transcription factors

DMI requires in input a list of target genes G for a TF of interest, a set of Gene Expression Profiles (GEPs) and a list of potential modulators M to test (Additional file 1: Figure S1). Therefore, in order to evaluate the performance of DMI when applied to real experimental data, we first collected *bona-fide* transcriptional targets from Chromatin ImmunoPrecipitation (ChIP) [10] and binding motifs data [11] for Transcription Factors (TFs) whose activity is regulated by a set of well-characterized kinases. We thus selected 14 TFs for which high quality information was available (Additional file 1: Table S3 and Material and Methods).

We then selected a compendium of 5,372 high quality human GEPs representing 369 different cells and tissue types, disease states and cell lines [8] (Material and Methods). To generate the list of potential modulators to test, we selected all of the 481 genes associated to a Gene Ontology (GO) molecular function term equal to “protein kinase activity” [12]. However, 190 out of 481 kinases had to be filtered out because their expression level was not changing sufficiently in the gene expression compendium, thus leaving a total of 291 kinases as potential modulators (Material and Methods).

We then applied the DMI method to the compendium of 5,372 GEPs for each of the 14 TFs. We thus obtained, for each TF, a list of the 291 kinases ranked according to their differential Multi-Information and with an associated *p*-value (Material and Methods).

**Fig. 2:**
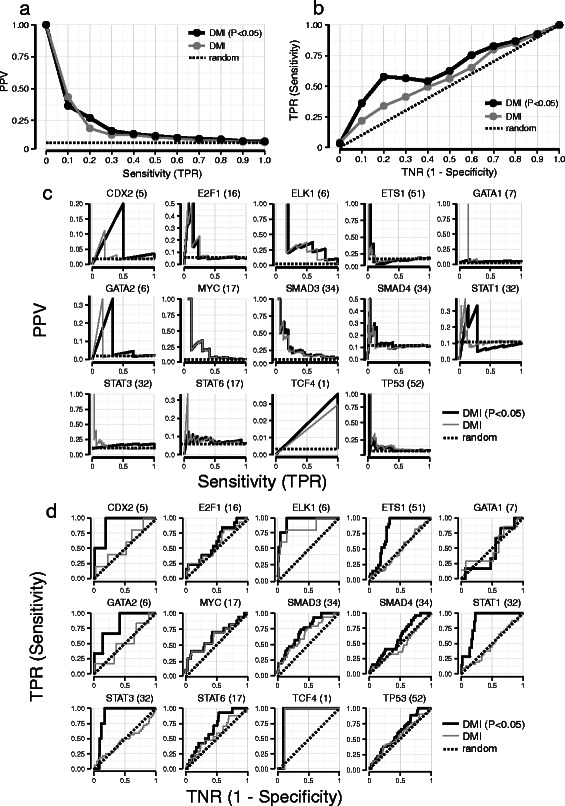
PPV-Sensitivity and ROC curves for 14 transcription factors. In parentheses the number of know kinases interacting with each TF present in the “Golden Standard”. A pre-filtering step based on the Fold Change (FC) of the modulator was applied to remove kinases with a FC ≤ 1 (Material and Methods). Positive Predicted Value (PPV) or precision is computed as a fraction of *TP*/ (*TP + FP*). Sensitivity (or true positive rate, TPR) is computed as a fraction of *TP*/ (*TP + FP*). True Negative Rate (TNR) is coputed as 1 – Specificity with Specificity equal to *TP*/ (*TP + FP*). **a** The cumulative PPV-Sensitivity curve of DMI across the 14 transcription factor obtained by averaging the individual PPV-sensitivity curves of each TFs (Material and Methods); **b** The cumulative receiver operating characteristic (ROC) curve of DMI across the 14 transcription factor (Material and Methods); **c** PPV-sensitivity curve for each one of the 14 transcription factor in which we compared the performance of DMI with and without applying a significance threshold for the *p*-value (P < 0.05) after Benjamini-Hochberg correction; **d** ROC curve for each one of the 14 transcription factor in which we compared the performance of DMI applying a significance threshold for the *p*-value (P < 0.05) after Benjamini-Hochberg correction

In order to assess the predictive ability of DMI, we collected the known kinases modulating the activity of each of the 14 TFs from PhosphoPOINT [13], NetworKIN [6] and CEASAR [7]. We thus obtained a “Golden Standard” for each TF consisting of experimentally verified kinases (Material and Methods). We estimated the performance of DMI by computing the both the overall PPV-Sensitivity and receiver operating characteristic (ROC) curves across the 14 TFs (Fig. 2a–b) and both the individual PPV-Sensitivity and ROC curves for each of the TFs (Fig. 2c–d and Additional file 1: Figure S4). We also reported the expected performance when ranking the 291 modulators randomly (dashed line in Fig. 2). It can be appreciated that the DMI performance is about ten-fold better that the random performance.

These results show that top-ranking kinases according to DMI are those that have a high probability of being the modulators for most of the TFs tested.

We also predicted for each transcription factor the kinase family regulating it, as well as the most likely signalling pathway controlling the TF activity. To this end, we detected whether members of a specific family of kinases or signalling proteins were statistically enriched at the top of the ranked modulators’ list as reported in Table 1 and Additional file 1: Table S1 (Material and Methods).

**Table 1:** Kinase subfamilies predicted by DMI to modulate the 14 TFs used for validation

TFs	Subfamily Predictions
CDX2	PIM, **MAPK [db]**, DMPK, **CDC2/CDKX [db]**, SYK/ZAP-70, Lammer, VRK
E2F	**MAPK [db]**, CSF-1/PDGF receptor, CaMK
ELK1	CSF-1/PDGF receptor (0.001), **MAPK [db]**
ETS1	**CaMK [db]**, HIPK, **MAPK [db]**
GATA1	CaMK, HIPK, **MAPKK** [24], GCN2, **MAPK [db]**
GATA2	CaMK, **MAPK [db]**, DMPK, **SRC** [25]
MYC	**CaMK** [26], **CSF-1/PDGF receptor** [27], **MAPK [db]**, HIPK, GCN2, **SRC** [28]
SMAD3	**DMPK [db]**, **CSF-1/PDGF receptor** [27], **MAPK [db]**, PIM, Lammer, **CaMK [db]**
SMAD4	**CaMK [db]**, DMPK, **MAPK [db]**, PIM, **HIPK** [29], GCN2, **SRC [db]**
STAT1	**CaMK [db]**, BUB1, STE20
STAT3	**CSF-1/PDGF receptor [db]**, DMPK, SYK/ZAP-70
STAT6	**EGF receptor** [30], **Fibroblast growth factor receptor** [31], I-kappa-B kinase, **CSF-1/PDGF receptor** [32], **MAPKKK** [33], **JAK [db]**, AXL/UFO
TCF4	**CaMK** [34], DMPK, **MAPK [db]**, PIM, HIPK
TP53	**CSF-1/PDGF receptor** [35], **Lammer [db]**, **MAPK [db]**, **DMPK [db]**

In bold, subfamilies correctly identified by DMI as confirmed either by literature or by a phospho-interactome database [db] (Material and Methods). Kinase subfamilies are sorted according to the *p*-value of their enrichment score and results have been cut with a *p*-value threshold of 0.01

### Comparison with MINDy

We compared the performance of DMI with MINDy [5] (Material and Methods), a state-of-the-art computational method for the identification of post-translational modulators from gene expression profiles. MINDy is based on a pair-wise computation of Mutual Information between the TF and each of its target genes, whereas DMI is based on an ensemble estimation of the Multi-Information across all of the target genes, without the need to assume that the TF is co-expressed with its target genes.

**Fig. 3:**
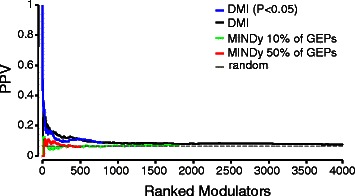
Comparison between MINDy and DMI for the identification of the post-translational modulators of 14 TFs. PPV (Positive Predicted Values) vs. Ranked Modulators plot for MINDy and DMI methods. DMI performance when selecting only the modulators with a fold-change greater than one (FC > 1) (*black line*), or when keeping only the predicted kinases with a p-value P < 0.05 (*blu line*). The expected performance of a random algorithm is 0.06 (*red dashed line*). Since the absolute value of ∆I is not strictly comparable among different TFs, because it also depends on the number of targets, we computed for each tested kinase a normalized ∆I value as: *ΔI* = (*I*_*HIGH*_ − *I*_*LOW*_)/(*I*_*HIGH*_ + *I*_*LOW*_)

We used MINDy to predict from the list of 291 kinases, the modulators of the 14 TFs. The PPV curve computed from MINDy predictions was compared to the one obtained by applying the DMI method (Fig. 3). Both methods performed better than random, but DMI has clearly an improved performance. It has to be taken into account, however, that DMI requires the knowledge of the TF target-genes, whereas MINDy automatically predicts, given the TF, its target genes, as well as, the post-translational modifiers of the TF activity. Hence, MINDy uses much less information than DMI, therefore a lower performance is to be expected. Additional file 1: Figure S5 shows the PPV curve for MINDy when forcing MINDy to use only the collected bona-fide targets for each one of the 14 TFs.

The results of the comparison show that the two approaches are complementary, in that if the targets of the TF are known, DMI offers a better predictive ability than MINDy; on the other hand if the targets are unknown, DMI cannot be applied, whereas MINDy is generally applicable.

## Discussion and conclusions

DMI is based on the assumption that when a post-translational modulator activates a transcription factor, its target genes will be co-expressed, and hence co-regulated (the opposite will happen if the modulator de-activates the TF). We further assume that the level of expression of the modulator is a good proxy for its activity in the cell. It is important to underline that our working hypothesis does not rely on changes in the TF expression level, nor of its target genes but rather on changes in their *co-regulation*. This is relevant, since we have previously shown that changes in the co-regulation of metabolic pathway enzymes are predictive of their tissue activity even when their expression levels are low and do not change significantly across tissues [14].

DMI relies on the estimation of the Renyi Multi-Information of the TF’s direct target genes in a subset of GEPs as a measure of the degree of their co-regulation. Unlike other common techniques that measure *pair-wise* co-regulation between genes, such as correlation and mutual information, Multi-Information can estimate co-regulation among all of the target genes at once. This property makes Multi-Information more robust than pair-wise approaches, thus reducing the number of false positives.

Our strategy, differently from the others proposed in the literature, does not require the transcription factor and its target genes to be co-expressed, thus making the approach more generic, albeit requiring the TF’s target genes to be known. We also performed additional analyses supporting our working hypothesis showing that in presence of a post-translational modulator of a TF, the TF itself does not necessarily change its expression level nor it correlates with its target genes (Additional file 1: Supplementary Data, sections 1.2 and 1.3 of “Additional analysis” and Additional file 1: Figures S6–S8).

We first showed that DMI is able to correctly identify post-translational modulators of 14 transcription factors including P53, MYC and members of STAT and SMAD families from a compendium of 5,372 GEPs [8].

One of the limitations of our approach is the assumption that the expression level of the modulator (e.g. a kinase or phosphatase) is a good proxy of its enzymatic activity, which may not always be the case. Moreover, we require that expression level of the modulator across the compendium of GEPs changes at least one-fold, otherwise no significant prediction can be made. A further limitation is that DMI needs in input a subset of the TF’s target-genes.

Despite these limitations, DMI can be effectively used for the identification of post-translational regulatory interactions affecting the activity of a transcription factor in an efficient and cost-effective manner, thus filling the gap between transcriptional networks, identified by classic reverse-engineering approaches, and signalling networks identified by ad-hoc experimental approaches.

## Methods

### Estimation of the Rényi Multi-Information

The Rényi Multi-Information (RMI) can be used to estimate the statistical dependency among *d* real-valued random variables **X** = (*X* ^1^, *X* ^2^, …, *X* ^*d*^) with joint probability density function *f* : *ℝ* ^*d*^ → *ℝ* and marginal densities *f*_*i*_ : *ℝ* → *ℝ*, 1 ≤ *i* ≤ *d* [9, 15]. For *α* ≠ 1, RMI is defined for any real parameter *α*, assuming the underlying integrals exist, as:

$$ {I}_{\alpha}\left(\boldsymbol{X}\right)={I}_{\alpha }(f)=\frac{1}{\alpha -1}{\displaystyle {\int}_{{\mathbb{R}}^d}\frac{f^{\alpha}\left({x}^1\dots {x}^d\right)}{{\left({\prod}_{i=1}^d{f}_i\left({x}^i\right)\right)}^{\alpha -1}}}d\left({x}^1\dots {x}^d\right) $$

When *α* = 1, *I*_*a*_ (***X***) is defined in the limit *I*_1_ = log_*a* → 1_ *I*_*α*_. Indeed, the classical *multi*-information across *d* variables is just a special case of RMI with *α* = 1. In what follows, we set *α* = 0.99.

As reported in [15] the RMI among the *d* real-valued random variables **X** = *X* ^1^, *X* ^2^, …, *X* ^*d*^ from a sample of i.i.d. random variables **X**_1 : *n*_ = **X**_1_, **X**_2_, …, **X**_*n*_, we adapted an algorithm based on the generalized nearest-neighbor graph with the copula transformation. First of all, the algorithm estimates the entropy *H*_*α*_(*f*) for *α* ∈ (0, 1) as follows:

$$ \widehat{H}\left({\mathbf{X}}_{1:n}\right)=\frac{1}{1-\alpha } \log \frac{L_p\left({\mathbf{X}}_{1:n}\right)}{\gamma {n}^{1-p/d}}\dots \mathrm{where}\kern0.24em p=d\left(1-\alpha \right) $$

where *L*_*p*_(⋅) equals to the sum of the *p*-th power of Euclidian distance of the nodes in the nearest-neighbor graph *NN*_*S*_(⋅) for some finite non-empty *S* ⊂ *ℕ* ^+^; *γ* is a numeric constant dependent on *d, p* and *S* that can be estimated empirically from a large sample (*n* ≫ 1) [15]. Finally, the Rényi Multi-Information *I*_*α*_ of the *d* variables **X** = *X* ^1^, *X* ^2^, …, *X* ^*d*^ from a sample of i.i.d random variables **X**_1 : *n*_ = (*X*_1_ … *X*_*n*_) can be computed as [15],:

$$ {\widehat{I}}_{\alpha}\left({\mathbf{X}}_{1:n}\right)=-{\widehat{H}}_{\alpha}\left({\widehat{\mathbf{Z}}}_1,{\widehat{\mathbf{Z}}}_2,\dots, {\widehat{\mathbf{Z}}}_n\right) $$

Where *Ĥ*_*α*_ is defined as before and the sample $\left({\widehat{\mathbf{Z}}}_1,{\widehat{\mathbf{Z}}}_2,\dots, {\widehat{\mathbf{Z}}}_{\mathrm{n}}\right)=\left(\widehat{\mathbf{F}}\left({\mathbf{X}}_1\right),\widehat{\mathbf{F}}\left({\mathbf{X}}_2\right),\dots, \widehat{\mathbf{F}}\left({\mathbf{X}}_{\mathrm{n}}\right)\right)$. $\widehat{\mathbf{F}}\left(\cdot \right)$ is called *empirical copula transformation* [16], where the *j*-th coordinate of **Ẑ**_*i*_ equals:

$$ {\widehat{Z}}_i^j=\frac{1}{n}\mathrm{rank}\left({X}_i^j,\left({X}_1^j,{X}_2^j,\dots, {X}_n^j\right)\right) $$

where rank (*x*, *A*) is the number of elements of *A* less than or equal to *x*.

The computational complexity *T*(*n*) for the estimation of Rényi Multi-Information strongly depends of the complexity of the K nearest-neighbors algorithm, which is linear in the number of points and the number of features for each point, and the complexity of copula transformation, which is quadratic in the number of points. Specifically, the computational complexity for the estimation of Rényi Multi-Information is:

$$ T(n)=O\left({n}^2d+nd\right) $$

where *n* is the number of i.i.d. samples used for its estimation (in this setting it represents the number of gene expression profiles) and *d* is the number of features of each i.i.d sample (i.e. number of target genes).

### Convergence of Rényi Multi-Information estimator (*Î*_*α*_)

We tested the convergence of the estimation algorithm to the true value of the Rényi Multi-Information numerically by generating simulated dataset of 4000 i.i.d. samples each, sampled from a multivariate Gaussian distribution of dimension *d* = 3, 10 or 20 with zero mean and an identity covariance matrix, corresponding either to independent variables (i.e. the true value is *I* = 0), or to a randomly chosen symmetric covariance matrix, corresponding to dependent variables (i.e. with an *I* > 0). The estimation of *Î*_*α* = 0.99_ among *d* = 3 variables and its error are shown in Additional file 1: Figure S9 in the case of dependent variables (i.e. *I* > 0), and in Additional file 1: Figure S10 in the case of independent variables (i.e. *I* = 0). Additional file 1: Figure S11 reports the estimation of *Î*_*α* = 0.99_ among 10 and 20 variables in both cases of dependent and independent variables.

We then repeated the same analysis as above, but this time generating simulated dataset of 4000 i.i.d. values sampled from a multivariate Beta distribution (rather than a Gaussian as before) of dimension 10 and 20 with alpha and beta parameter randomly selected from the standard uniform distribution in the open interval [0,1]. Additional file 1: Figure S12 shows the estimation of *Î*_*α* = 0.99_ among 10 and 20 variables in the case of dependent and independent variables. The Gaussian Copula transformation was used to build these distributions. For more details and the closed-form expression of the true divergence with Beta distribution please refer to [17] (lemma 14).

### Differential Multi-Information method (DMI)

The DMI method is based on quantifying the change in *co-regulation* among a set of downstream targets *G* ^1^ … *G* ^*d*^ of a TF in the presence or absence of a modulator *M*, by estimating the difference in Renyi Multi-Information between two subsets of GEPs. These subsets are obtained by first sorting GEPs according to the expression of the modulator M being tested and then dividing the ranked list of GEPs into two (or more) subsets. A pre-filtering step is applied to remove those modulator genes (M) whose expression does not change significantly between the “high” subset (i.e. where M is highly expressed) and the “low” subset (i.e. M is expressed at low levels). Specifically, we excluded from the analysis those modulators whose average expression in the “high” subset divided but their average expression in the “low” subset (i.e. the fold change) is less than one.

### Computation of the Significance of ∆I using permutation tests

We used a permutation test in order to estimate the empirical distribution of ∆**I** and, from that, the associated p-value. Specifically, given a set of *d* target genes (i.e. variables), we computed the significance of a modulator *M* by randomly selecting *d* genes in *L* = 10,000 number of trials, and each time computing the ∆**I** value thus obtaining its empirical distribution. The *p*-value was finally estimated as the percentage of random trials with a value of ∆**I** greater than the measured one.

### Construction of the “in silico” dataset D1 and D2

In order to construct the in silico datasets D1 (and similarly for D2) we simulated two sets of gene expression profiles. One set (co-regulated set) was obtained by sampling from a multivariate Gaussian distribution with zero mean and a covariance matrix whose elements were equal to *ρσ*_*ij*_^2^, where *ρ* = 0.6 and *σ*_*ij*_^2^ randomly chosen in the interval ]0, 0.5[. The second set (independent set) was obtained by changing the covariance matrix to a diagonal matrix with *σ*_*ii*_^2^ randomly chosen in the interval ]0, 0.5[. The expression profiles of the *potential modulators* (i.e. modulators that do not regulate the TF) were generated using a Gaussian distribution with zero mean and variance *σ* ^2^ in the interval ]0, 0.5[. Finally, in order to simulate the expression profiles of the *effective modulators* we followed this strategy: in the co-regulated subset, we sampled from a Gaussian distribution *N* (1,0.1) (i.e. with average expression equal to 1), on the contrary in the independent subset, we used a normal distribution *N* (1,0.1) (i.e. with average expression equal to 0).

### Gene expression profile compendium and kinase selection

We applied DMI to a compendium of 5,372 high quality human GEPs representing 369 different cell and tissue types, disease states and cell lines, described in [8]. GEPs were measured using the Affymetrix HG-U133A platform. We normalized this dataset using the Robust Multi-array Average (RMA) normalization as implemented in the R package Bioconductor [18] and using the custom CDF files present on BrainArray [19], thus obtaining a gene-wise normalised dataset.

The list of 481 kinases to test as possible modulators for a given transcription factor was obtained by collecting all the genes with an associated Gene Ontology (GO) molecular function term equal to “protein kinase activity”. Only 291 out 481 kinases were used in further analyses, because only 291 out of 481 kinases had a fold change greater than one in the GEP compendium.

### Gene Set Enrichment Analysis for the prediction of kinases’ family and signalling pathways

Gene Set Enrichment Analysis (GSEA) [20] was applied to the ranked list produced by DMI to identify kinase subfamilies and signalling pathways regulating the TF activity. We downloaded the information regarding the kinase subfamilies from a recent published study collecting a total of 40 distinct subfamily [21] (Additional file 1: Table S2). In order to apply the GSEA, we used only subfamilies with more than one member. We also collected 22 signalling pathways from MSigDb (the curated dataset CP:KEGG) [11] (Additional file 1: Table S3).

### Comparison with MINDy

MINDy is computationally intensive and requires a large amount of memory due to large number of samples in our GEP compendium [5]. Thus, before running MINDy we had to reduce the number of samples. To this end, we built two dataset containing the 10 % and the 50 % randomly selected samples from the compendium of 5,372 GEPs. We run MINDy using the default parameters. In the first step MINDy computes Mutual Information (MI) between a modulator and transcription factor (TF) to test the statistical independence between them. Once statistical independence between modulator and TF pair is established, MINDy ranks all samples from low to high expression of that modulator and selects 35 % of samples from each tail (low and high expression samples). In each tail, MINDy computes the mutual-information (MI) between the TF and all of its candidate target genes (MI_low_ and MI_high_) and it assesses the statistical significance of both MI values. If at least one of the two MIs is significant then MINDy calculates ΔMI, defined as ΔMI = MI_high_−MI_low_.

We assessed the statistical significance of ΔMI using a null model that is generated by randomising the data [5]. A TF-target pair is considered to be modulated by that modulator if the (corrected) *p*-value of ΔMI is < =0.05. Finally MINDy summarizes the result for each modulator pair by counting the number of target genes by that pair. Further details can be found in the original publication describing MINDy [5].

### Estimation of the cumulative PPV-Sensitivity and ROC curves

For the estimation of the composite PPV-Sensitivity (or Precision-Recall) curve across the 14 transcription factors the tecnique of the 11-point interpolated average precision [22] was used. Basically, for each transcription factor the interpolated PPV is measured at the 11 sensitivity levels of 0.0, 0.1, 0.2, 0.3, 0.4, 0.5, 0.6, 0.7, 0.8, 0.9 and 1.0. By definition the interpolated PPV *p*_*interp*_ at a certain sensitivity level *r* is defined as the highest PPV found for any sensitivity level *r* ' ≥ *r*: *p*_*interp*_(*r*) = *max*_*r* ' ≥ *r*_(*r* ') [22]. To notice, that with this definition, the interpolated PPV at a sensitivity of 0 is always defined as 1. Finally, the composite PPV-Sensitivity curve among the 14 TFs was estimated as the arithmetic mean across the 11 sensitivity levels of the interpolated PPV of each transcription factor.

For the estimation of the composite Receiver Operator Characteristic (ROC) curve the tecnique of the vertical averaging [23] was instead used. The vertical averaging consits in taking vertical samples of the ROC curves for fixed true negative rates (TNR) and averages the corresponding values of sensitivity. Specifically, 11 TNR levels of 0.0, 0.1, 0.2, 0.3, 0.4, 0.5, 0.6, 0.7, 0.8, 0.9 and 1.0 were used for the estimation of the composite ROC curve among the 14 TFs. Obviously one of more of the 11 TNR levels may be absent in some of the ROC cuvers we are vertical averaging, in these cases the corrispondig value of sensitivity to average has been simply estimated by interpolation using its next and prcedent value in the considered ROC curve.

## Additional file

Additional file 1:
**Supplementary Data.** Supplementary analysis and supplementary figures and tables. (DOCX 2030 kb)
