# Supplementary material for: A reverse-engineering approach to dissect post-translational modulators of transcription factor’s activity from transcriptional data
Source: BMC Bioinformatics. 2015 Sep 3;16:279. doi: 10.1186/s12859-015-0700-3 (PMC4559297; doi:10.1186/s12859-015-0700-3)
Supplement: Additional file 1: — Supplementary Data. Supplementary analysis and supplementary figures and tables. (DOCX 2030 kb) [file 12859_2015_700_MOESM1_ESM.docx]

SUPPLEMENTARY DATA

Table of Contents

1. Additional Analysis 2

1.1 Other approach for the identification of PTMs and comparison with DMI 2

1.2 TFs expression in presence/absence of the Modulators 3

1.3 Kinase modulation of the target genes 3

2. Supplementary Figures and Legends 4

3. Supplementary Tables and Legends 11

4. References 14

# 1. Additional Analysis

## 1.1 Other approach for the identification of PTMs and comparison with DMI

The approach based on difference in Multi-Information (DMI) we used in this work is certainly not the only possible way to measure changes in the dependence among variables and hence to identify post-translational modulators of TF activity.

As we formalized in the Material and Method section, we are interested in detecting changes in co-regulation among the targets $G^{1}\cdots G^{i}$ of a TF due to a modulator$M$ (Figure 1). An alternative strategy to DMI could be represented by the use of a multidimensional independence test between the variable $\mathbf{G}=(G^{1}\cdots G^{i})$ and the variable $\mathbf{M}=(M^{1}\cdots M^{j})$ representing the modulators (i.e. here $\mathbf{M}=(M^{1})$). This is equivalent to consider a sample of $\mathbb{R}^{i}\times\mathbb{R}^{j}$ valued random vectors $\left( G_{1},M_{1} \right)\ldots\left( G_{n},M_{n} \right)$ with independent and identically distributed (i.i.d.) pairs defined on the same probability space and testing the null hypothesis ($H_{0}$) that $\mathbf{G}$ and $\mathbf{M}$ are independent:

$$H_{0}:P\left( \mathbf{G},\mathbf{M} \right)=P\left( \mathbf{G} \right)\times P(\mathbf{M})$$

possibly making minimal assumptions regarding the probability distributions of the variables. To test $H_{0}$ we can use one of the two methods presented in [[1](#_ENREF_1)], where the authors proposed two different approaches to test the independence between two multidimensional variables. The first method consists in partitioning the underlying space, and in evaluating a test statistics on the resulting discrete empirical measures. We did not test this method because of the computational complexity, which is exponential in the number of bins used for the discretization step elevated to the sum of the dimensions of the two variables to test. The method that we tested is the method based on kernel-density estimation. Obviously a limit of this strategy (i.e. test only the independence between two multidimensional variables), we confirmed in our tests, consists in the fact that if the modulator M being tested is not a real modulator but instead it is a itself a target of the TF, then it will be strongly co-regulated with the targets G, and the method would detect a dependence between M and G, and hence M would be flagged as a modulator of the TF. This modulator however would obviously be a false positive.

Another possible approach for the identification of PTMs could be of using the Conditional Multidimensional Independent Test (CMIT) described in [[2](#_ENREF_2)]. Here, the authors present a new measure of conditional dependence among random variables, based on normalized cross-covariance operators and on reproducing kernel Hilbert spaces. The CMIT test applied to the problem of finding modulator M of a TF can be implemented as follows: given the target genes$\mathbf{G}=(G^{1}\cdots G^{i})$ of the transcription factors $F$ and a modulator $M$, we can test the conditional independence of $\mathbf{G}$ and $F$ given $M$ using the CMIT test, thus solving the problem of finding the modulator for which the targets and the transcription factor(s) are co-regulated. To apply the CMIT test, we have to know which is the TF that regulates our targets (this condition is usually satisfied) but we also have to assume that the TF and targets are co-regulated and so statistically dependent, introducing others constraints in our model.

The comparison among the three methods $\boldsymbol{\Delta I}$**,** MIT and CMIT are reported in Supplementary Figure 13, where we show that the $\boldsymbol{\Delta I}$ perform significantly better than the other two.

## 1.2 TFs expression in presence/absence of the Modulators

In this work we assumed that the expression level of the transcription factor (TF) is not required for ranking candidate modulators. We verified this hypothesis showing how the TF expression distribution seems not to be influenced by the presence or absence of its modulators. We computed for each of the 14 TFs in the Golden Standard, two expressions distributions, one when the true modulator is highly expressed and the other when the true modulator is expressed at low levels. In a scenario where in the presence/absence of the modulator, the expression of the TF is altered, the two expression distributions will be well separated. As we can observe in Supplementary Figure 6, this happens for some TFs (i.e. ELK1, E2F1 ans GATA2) but this is not a generic property.

## 1.3 Kinase modulation of the target genes

In order to verify that that true kinase modulators tend to get higher DMI scores when compared with other random genes, we compared the average DMI scores among the targets genes of the 14 TFs, computed when considering the true kinase modulators present in the “Golden Standard”, with the average DMI computed using as possible modulator random genes. To this end, for each transcription factor 500 random genes (i.e. for a total of 500*14=7000 random gene tested) were tested as possible modulators. The results are summarized in the Supplementary Figure 7.

As comparison, we also verified how an approach based on a pair-wise similarity measure behaves. In particular we computed the average spearman correlation among the 14 TFs tested in the main text and their target when its true modulators in the “Golden Standard” are “up” or “down”. The results presented in Supplementary Figure 8 shows how there is no substantial difference between the average correlation in presence or absence of the True modulators of a TF.

# 2. Supplementary Figures and Legends


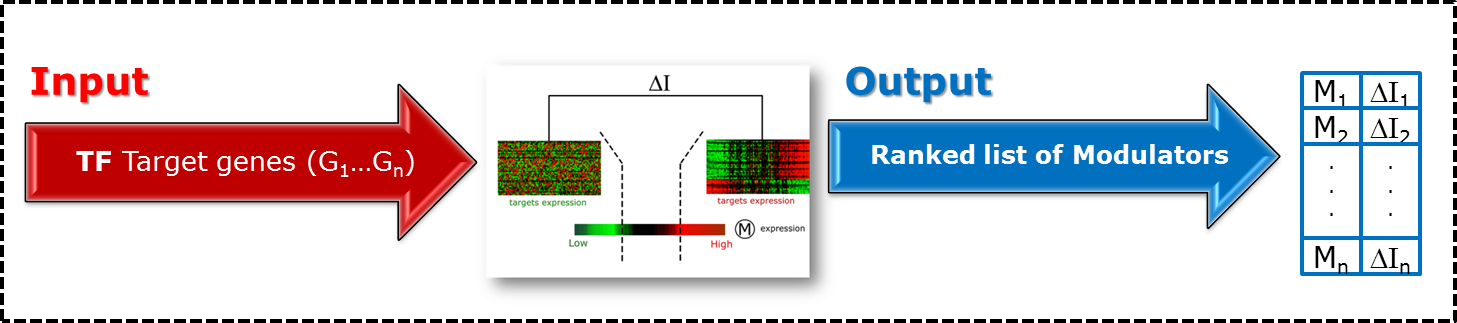


**Supplementary Figure 1** – The input to DMI is a set of targets (G_1_...G_n_) for the transcription factor of interest (TF). The output of DMI is a ranked list of possible modulators (M_1_...M_k_) regulating the TF activity. The modulators are sorted according to their ($\boldsymbol{\Delta I}$) which quantifies the ability of the modulator to influence the activity of the downstream targets of TF.


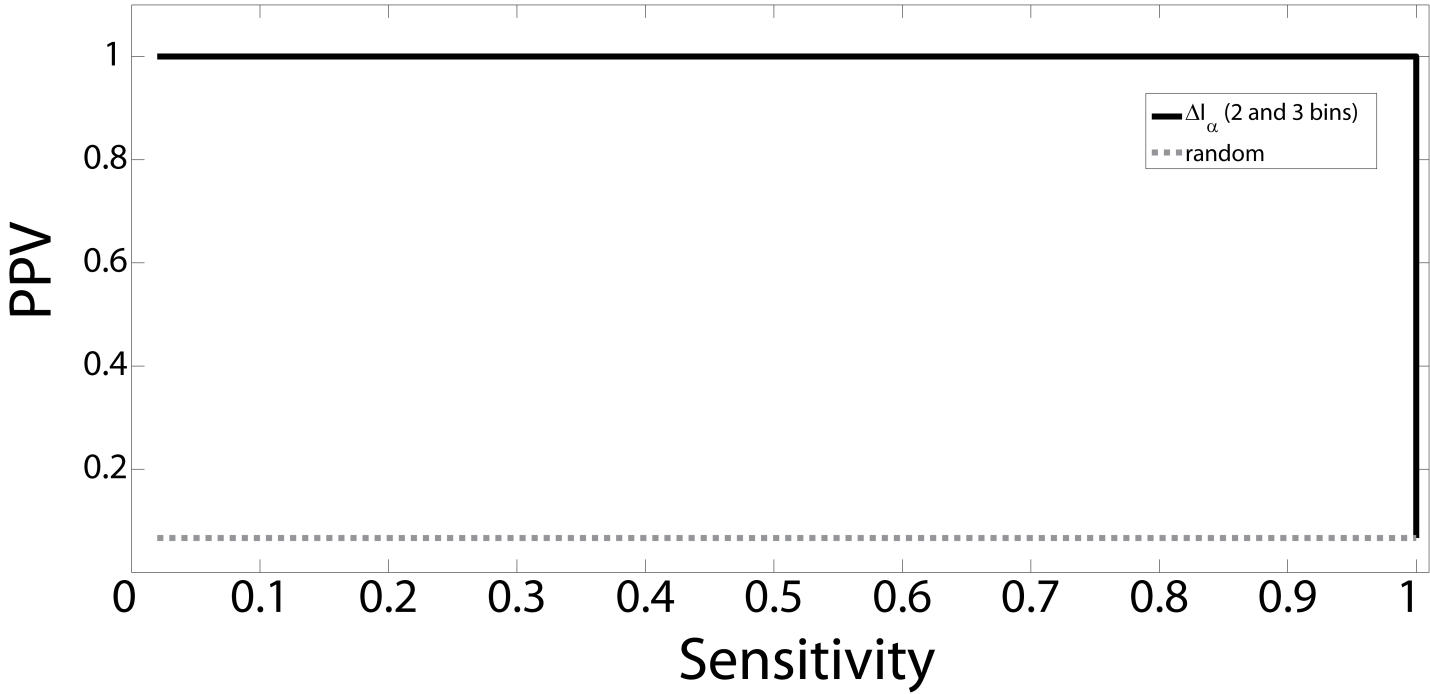


**Supplementary Figure 2** – DMI method performance in the “in silico” dataset D1. The PPV-sensitivity curveis reported when using only 2 . Only modulators with p-value = 0 have been selected. The random performance (dashed line) corresponds to an algorithm which randomly ranks the modulators.


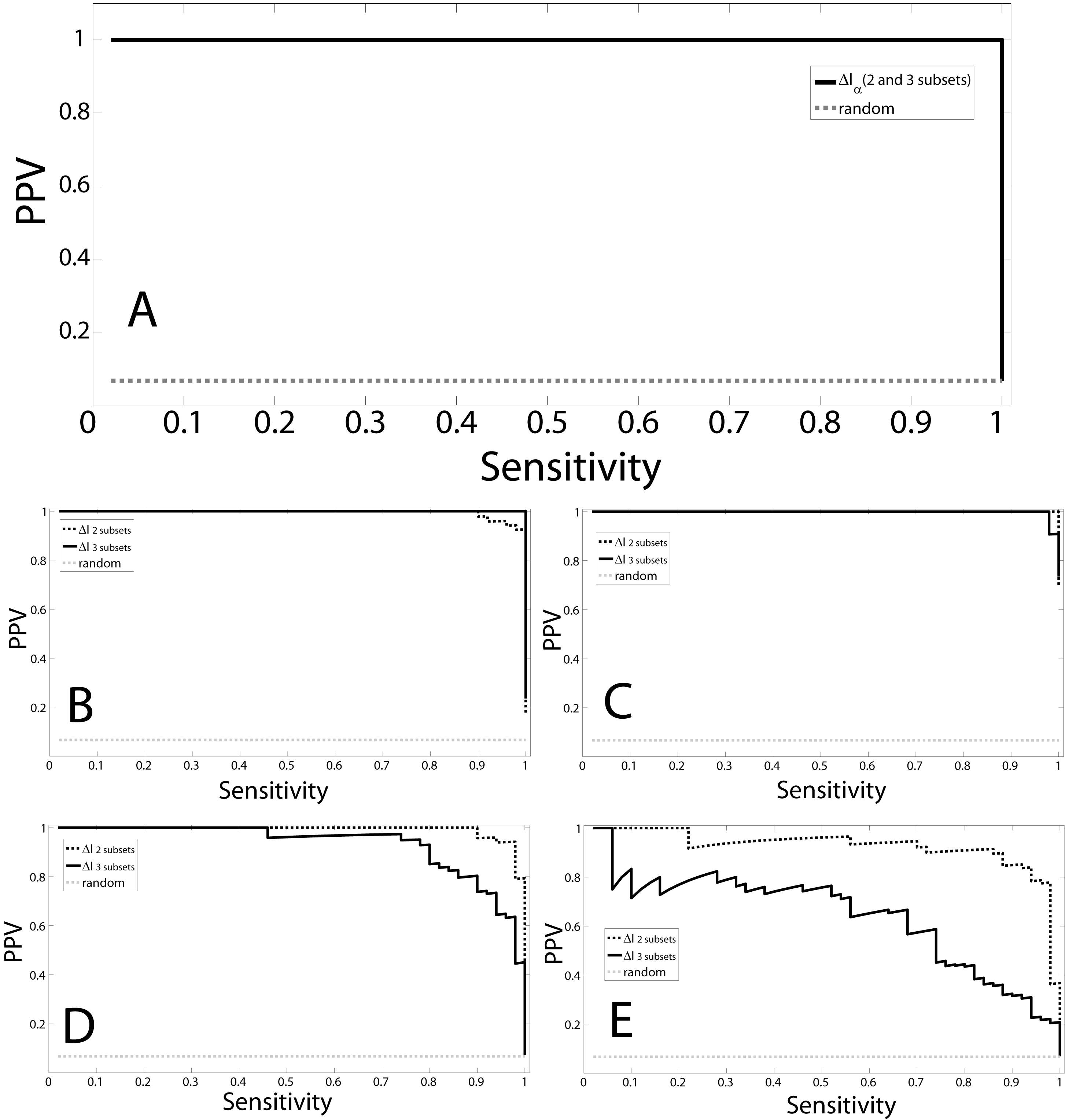


**Supplementary Figure 3 -** DMI method performance in the “in silico” dataset D2. The PPV-sensitivity curve when the targets are co-regulated in 50(**a**), or in 30 (**b**), 40 (**c**), 60 (**d**) and 70 (**e**) out of the 100 GEPs are shown. Only modulators with p-value = 0 have been selected. The random performance (straight dashed line) corresponds to an algorithm, which randomly ranks the modulators.


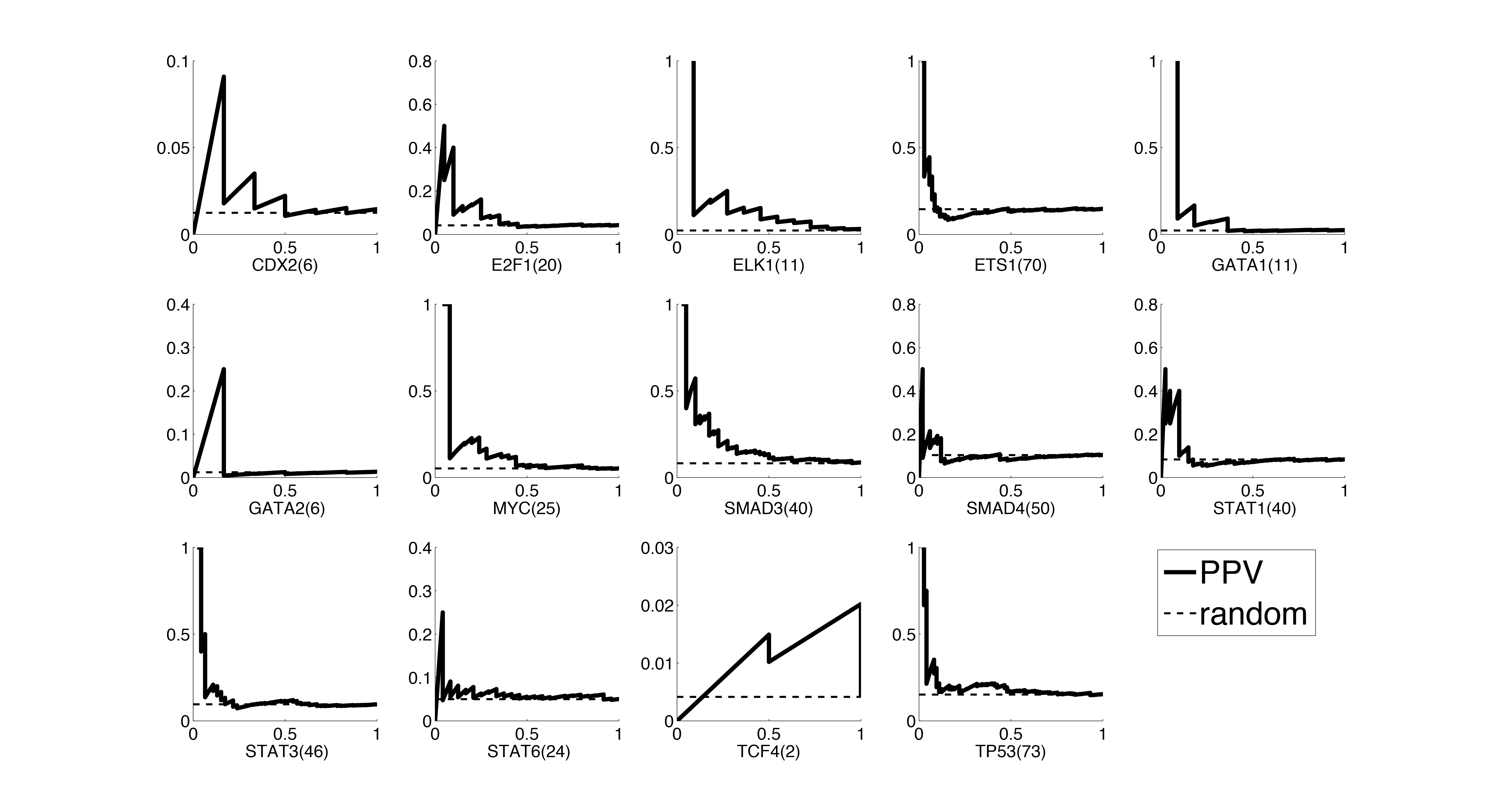


**Supplementary Figure 4** - PPV-sensitivity curve for 14 transcription factors without apply the pre-filtering step based on the Fold Change (FC) to remove kinases with a FC≤1 (Material and Methods).


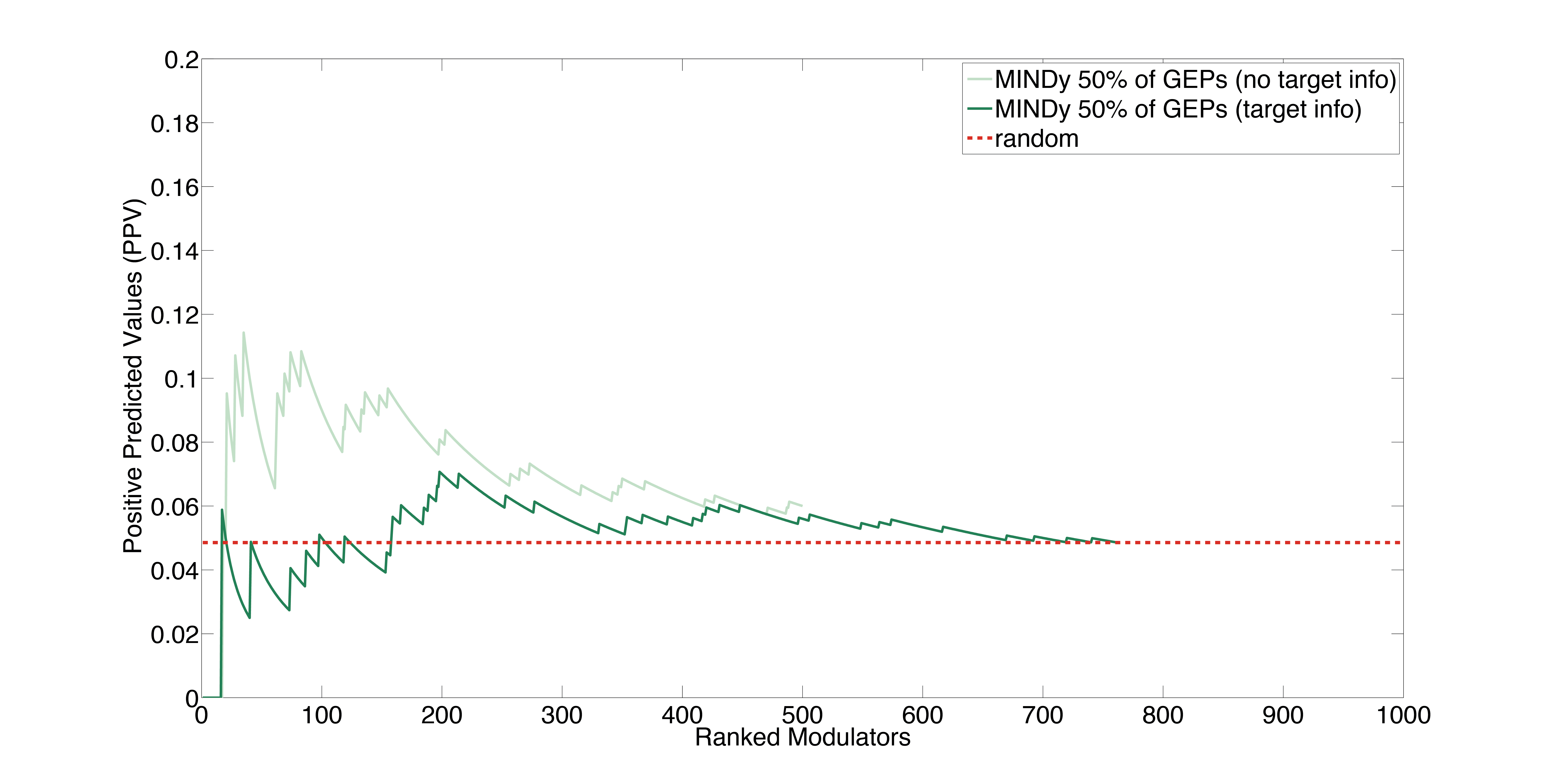


**Supplementary Figure 5 -** MINDy method performance for the identification of the post-translational modulators of 14 TFs. PPV (Positive Predicted Values) vs. Ranked Modulators plot. The expected performance of a random algorithm (red dashed line) is also reported for comparison.


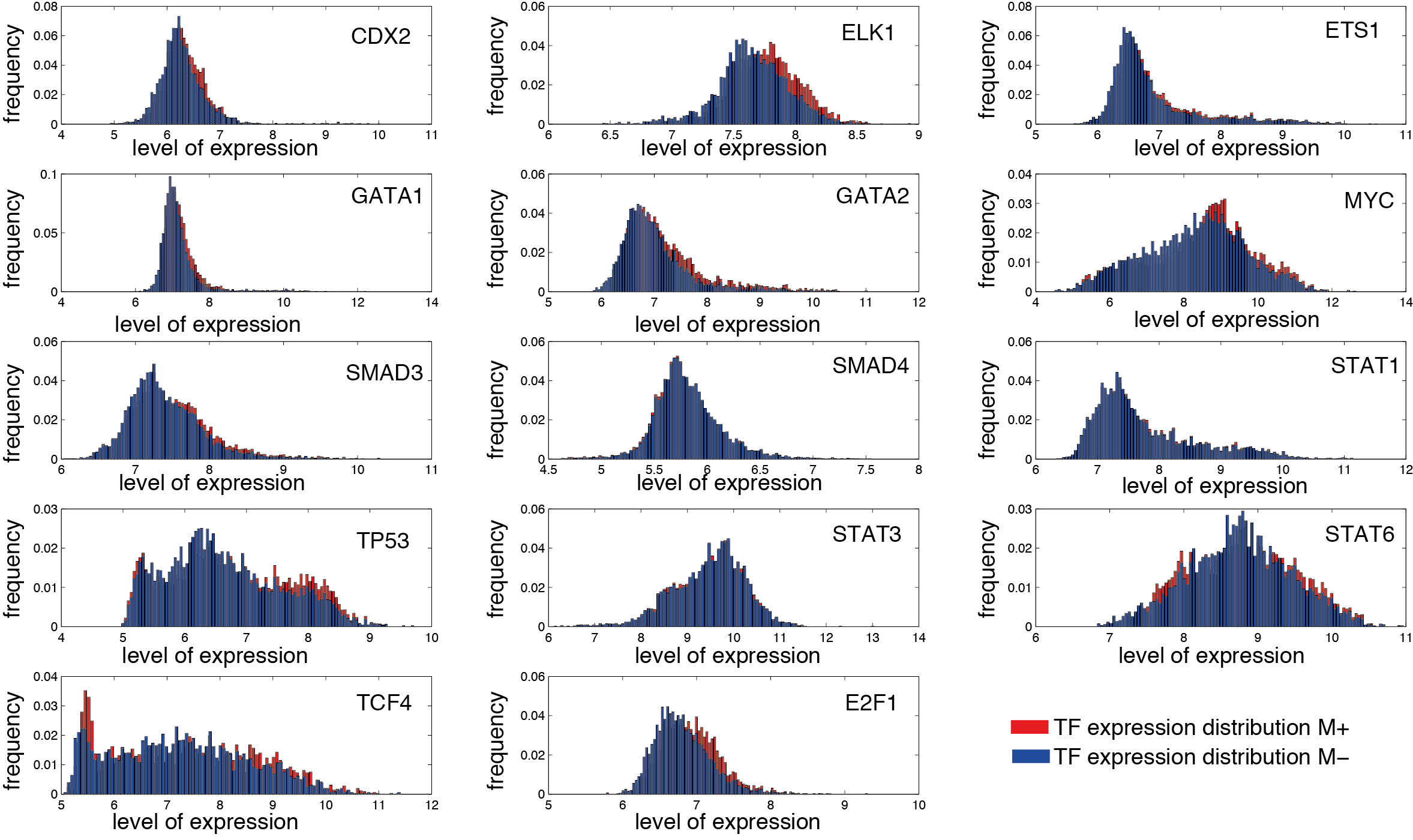


**Supplementary Figure 6** – TF distribution when the true modulators present in the golden standard are expressed at high (M+) or low (M-) levels.


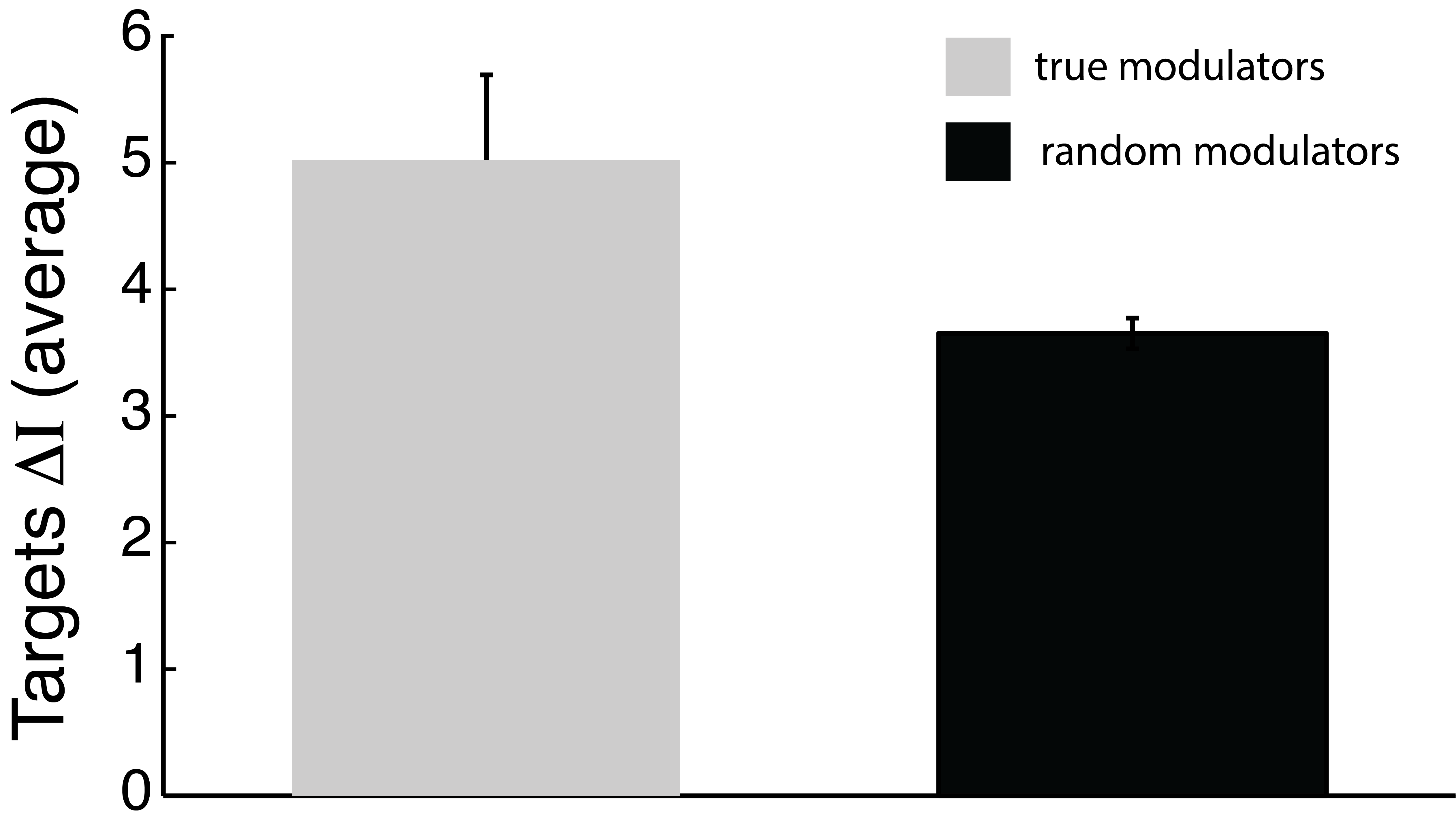


**Supplementary Figure 7** – Comparison between the average DMI among the targets genes of the 14 TFs tested for their true modulators present in the “Golden Standard” and the average DMI using as possible modulator random genes.


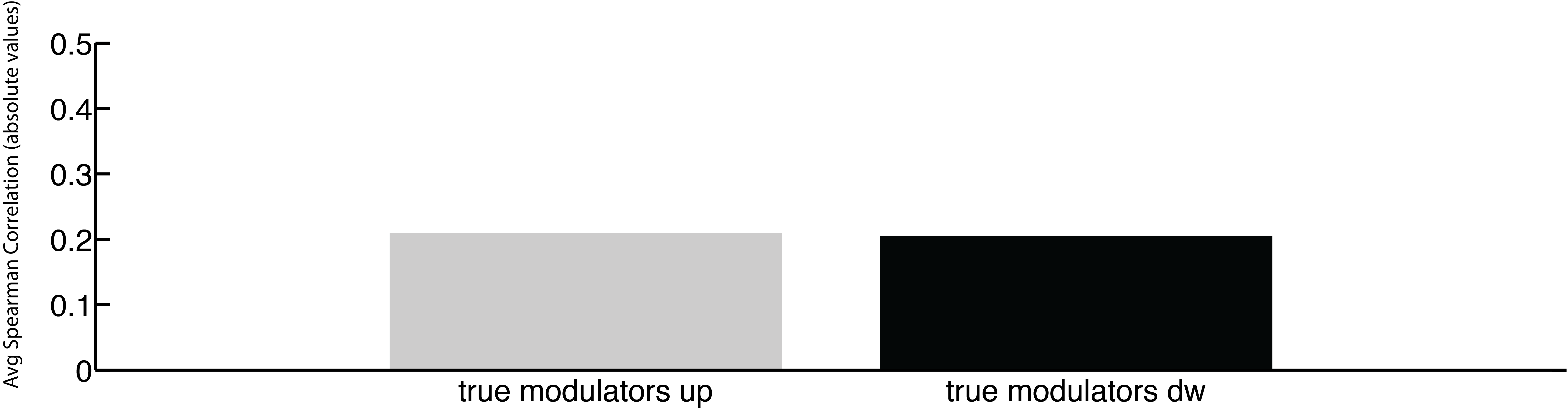


**Supplementary Figure 8** – Comparison between the average spearman correlation (in absolute values) between the target genes and each one of the 14 TFs tested. The averege correlation has been computed in the samples where the true modulators, present in the “Golden Standard”, are up (**a**) or down (**b**) (dividing the modulators expression in three equal bins).


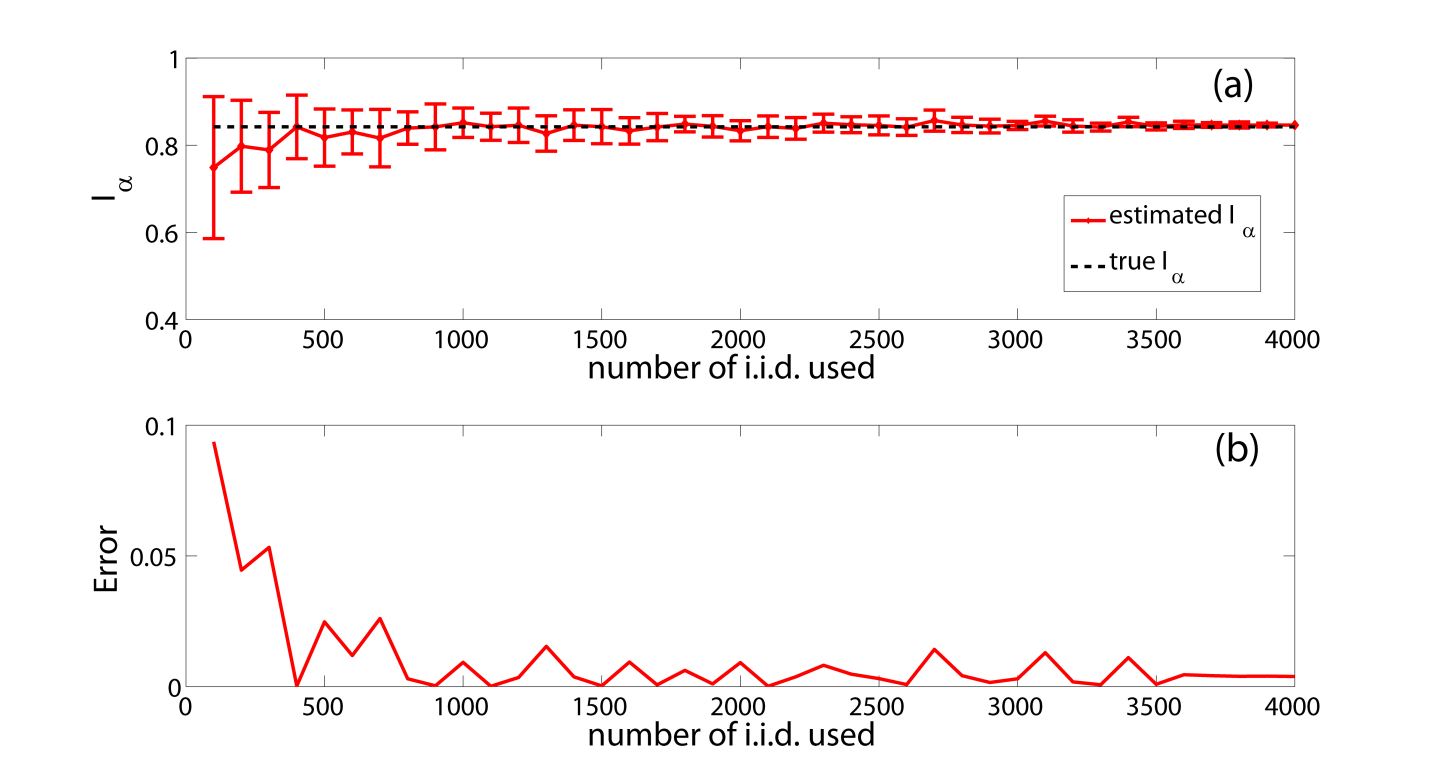


**Supplementary Figure 9 -** ${\hat{\boldsymbol{I}}}_{\boldsymbol{\alpha}}$ for 3 variables as a function of the number of i.i.d samples used for its computation. The 3 variables are dependent variables. The estimation of ${\hat{\boldsymbol{I}}}_{\boldsymbol{\alpha}}$ is computed 20 times for each point and its standard deviation is reported. **(a)** The convergence of ${\hat{\boldsymbol{I}}}_{\boldsymbol{\alpha=0.99}}$ to the true value of$\boldsymbol{I}_{\boldsymbol{\alpha}}$. **(b)** The error in the estimated value of ${\hat{\boldsymbol{I}}}_{\boldsymbol{\alpha=0.99}}$.


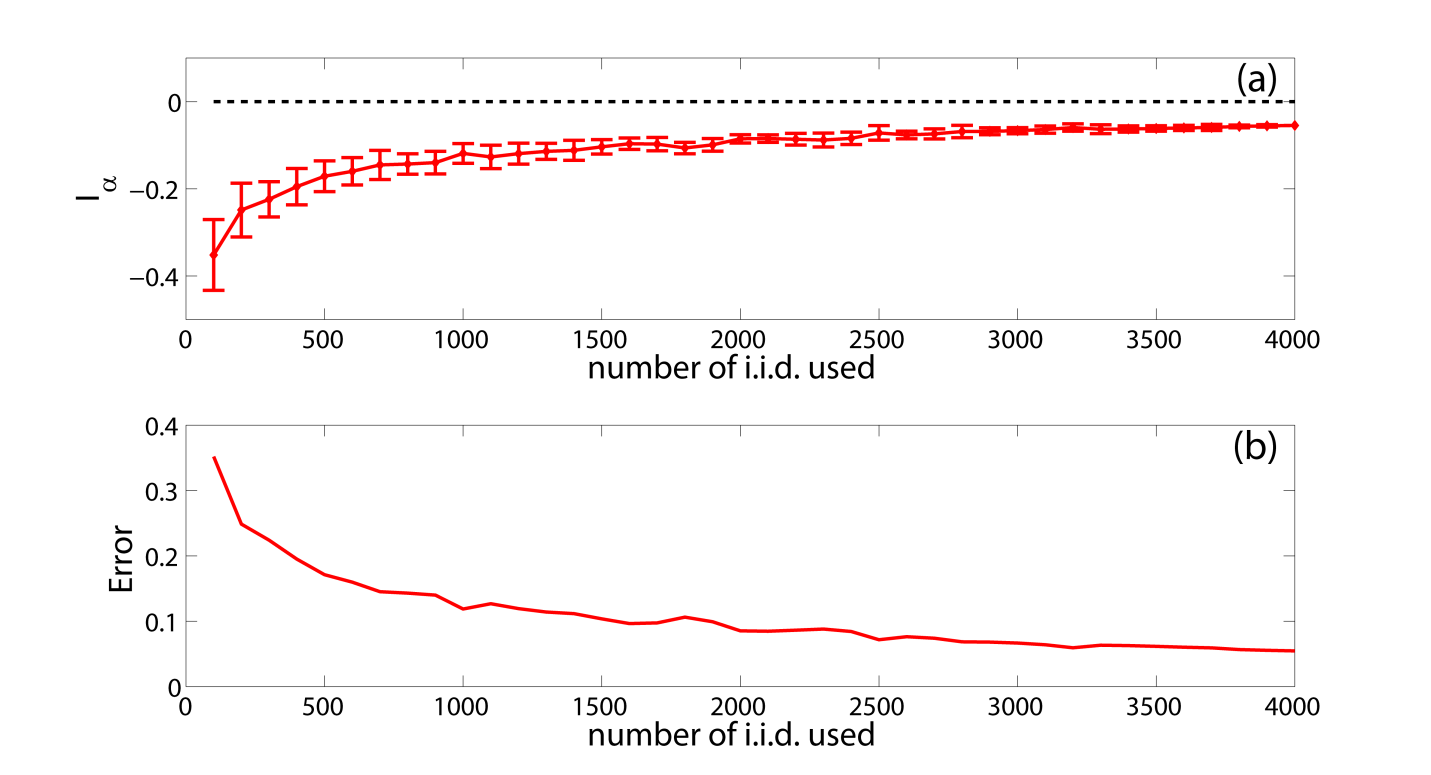


**Supplementary Figure 10** - ${\hat{\boldsymbol{I}}}_{\boldsymbol{\alpha}}$ among 3 variables as a function of the number of i.i.d samples used for its computation. The 3 variables are independent variables. The estimation of ${\hat{\boldsymbol{I}}}_{\boldsymbol{\alpha}}$ is computed 20 times for each point and its standard deviation is reported. **(a)** The convergence of ${\hat{\boldsymbol{I}}}_{\boldsymbol{\alpha=0.99}}$ to the true value of$\boldsymbol{I}_{\boldsymbol{\alpha}}\boldsymbol{(0)}$. **(b)** The error in the estimated value of ${\hat{\boldsymbol{I}}}_{\boldsymbol{\alpha=0.99}}$


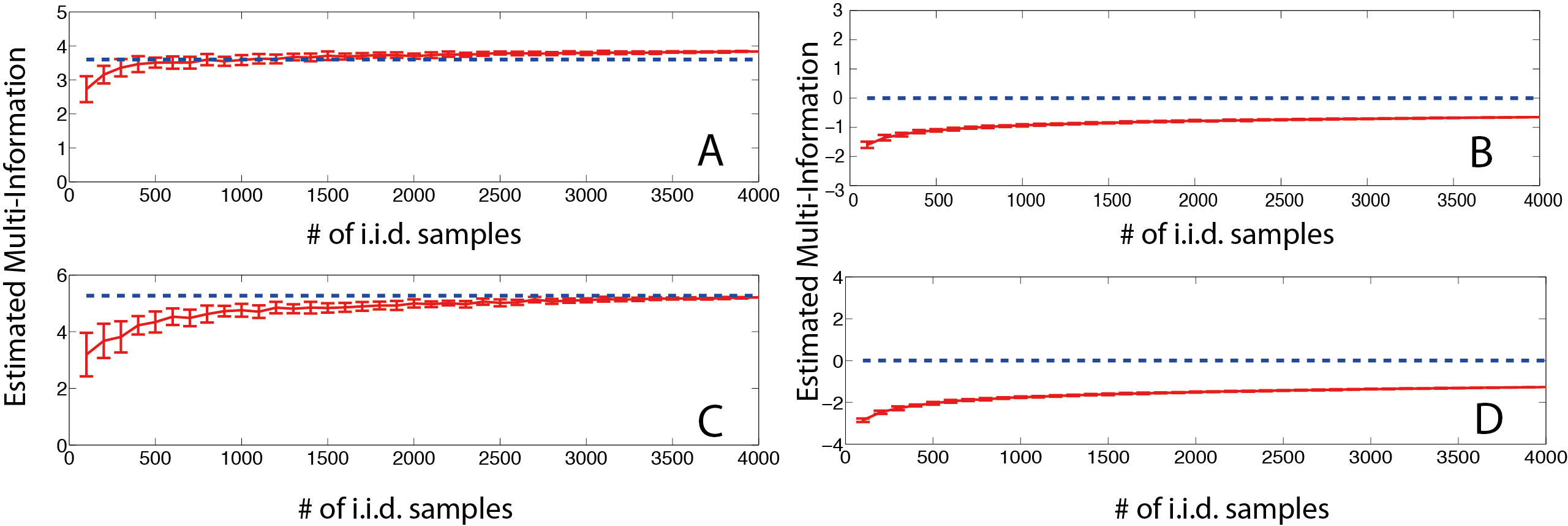


**Supplementary Figure 11** - ${\hat{\boldsymbol{I}}}_{\boldsymbol{\alpha=0.99}}$ among 10 and 20 variables generated from a multivariate Gaussian distribution as a function of the number of i.i.d samples used for its computation. The estimation of ${\hat{\boldsymbol{I}}}_{\boldsymbol{\alpha}}$ is computed 20 times for each point and its standard deviation is reported. (**a**) The 10 variables are dependent variables. (**b**) The 10 variables are independent variables. (**c**) The 20 variables are dependent variables. (**d**) The 20 variables are independent variables


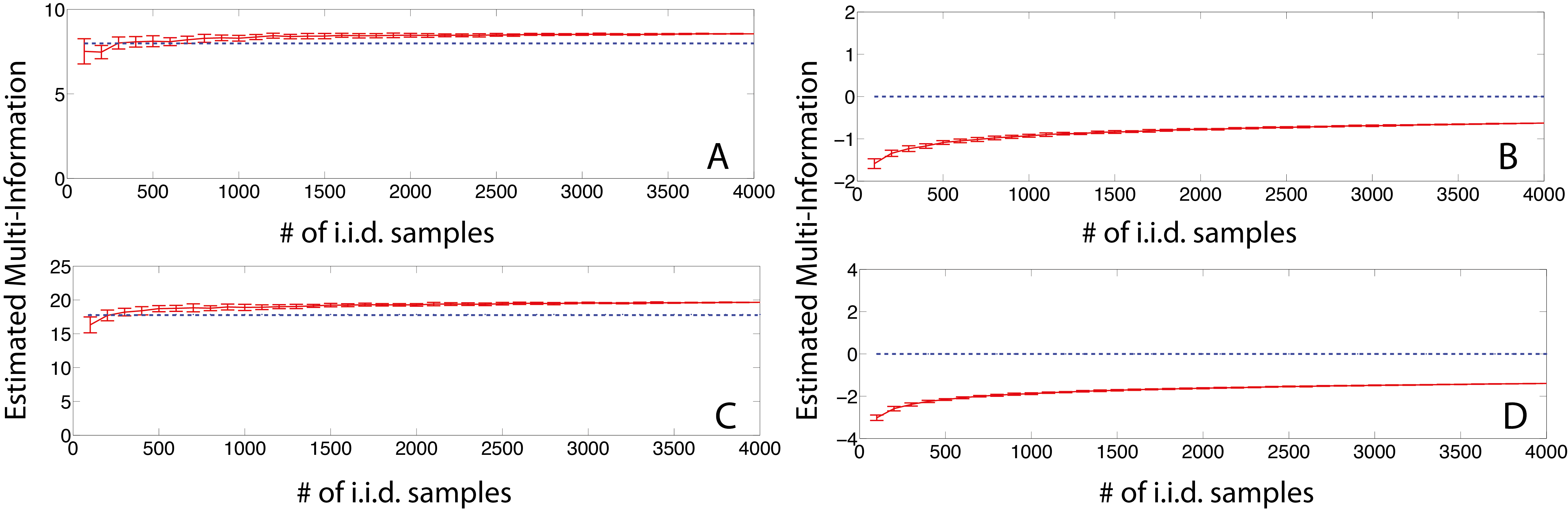


**Supplementary Figure 12** - ${\hat{\boldsymbol{I}}}_{\boldsymbol{\alpha=0.99}}$ among 10 and 20 variables generated from a multivariate Beta distribution as a function of the number of i.i.d samples used for its computation. The estimation of ${\hat{\boldsymbol{I}}}_{\boldsymbol{\alpha}}$ is computed 20 times for each point and its standard deviation is reported. (**a**) The 10 variables are dependent variables. (**b**) The 10 variables are independent variables. (**c**) The 20 variables are dependent variables. (**d**) The 20 variables are independent variables.


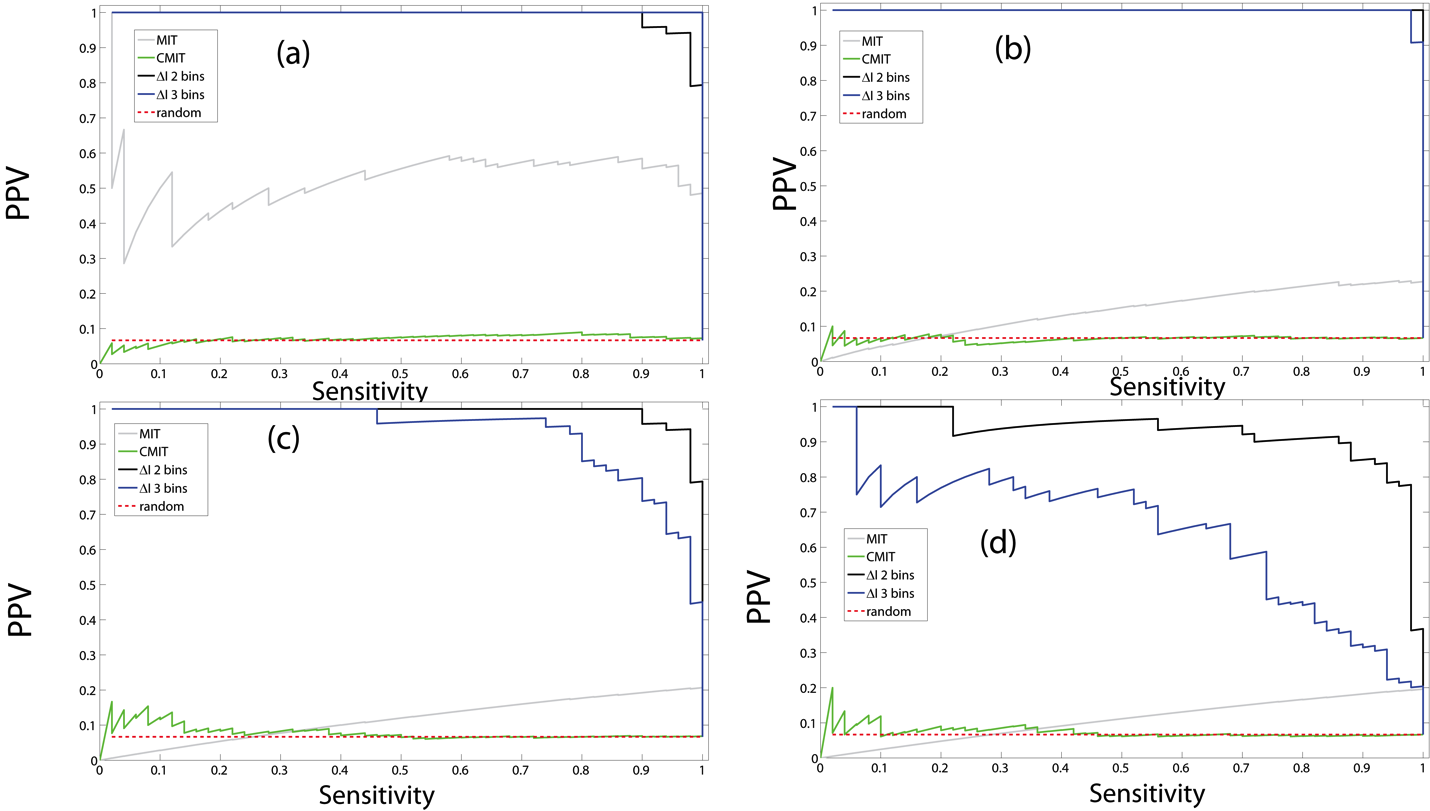


**Supplementary Figure 13** – Comparison among DMI and other two possible approach MIT and CMIT discussed in the supplementary data. PPV-sensitivity curve using “in-silico” dataset D2 where the targets are dependent in the 30 **(a)**, 40 **(b)**, 60 **(c)** and 70 **(d)** of the experiments.

# 3. Supplementary Tables and Legends

**Supplementary Table 1** – Enriched signaling pathway for the 14 TFs. In parentheses the p-value of the enrichment score computed with GSEA.

| **Official**  **Symbol** | **Signalling Pathways** |
| --- | --- |
| CDX2 | ERBB (0.0000), WNT (0.0050), INSULIN (0.0210), CHEMOKINE (0.0240) |
| E2F1 | GNRH (0.0000), FC EPSILON RI (0.0010), WNT (0.0030), VEGF (0.0040), MAPK (0.0110), T CELL RECEPTOR (0.0150), TOLL LIKE RECEPTOR (0.0170), NOD LIKE RECEPTOR (0.0190) |
| ELK1 | WNT (0.0000), GNRH (0.0000), FC EPSILON RI (0.0010), VEGF (0.0030), T CELL RECEPTOR (0.0070), TOLL LIKE RECEPTOR (0.0130), MAPK (0.0150), RIG I LIKE RECEPTOR (0.0320) |
| ETS1 | ERBB (0.0050), VEGF (0.0060), GNRH (0.0070), INSULIN (0.0120), T CELL RECEPTOR (0.0180), P53 (0.0220), WNT (0.0230) |
| GATA1 | MAPK (0.0000), FC EPSILON RI (0.0000), GNRH (0.0000), TOLL LIKE RECEPTOR (0.0020), VEGF (0.0030), T CELL RECEPTOR (0.0050), NEUROTROPHIN (0.0050), MTOR (0.0060), ERBB (0.0080), WNT (0.0140), PHOSPHATIDYLINOSITOL SIGNALING SYSTEM (0.0200) |
| GATA2 | ERBB (0.0000), GNRH (0.0020), WNT (0.0030), CALCIUM (0.0250) |
| MYC | GNRH (0.0000), VEGF (0.0010), FC EPSILON RI (0.0020), WNT (0.0120), T CELL RECEPTOR (0.0150), ERBB (0.0160), MAPK (0.0240), TOLL LIKE RECEPTOR (0.0320) |
| SMAD3 | GNRH (0.0050), VEGF (0.0090), TGF BETA (0.0130), ERBB (0.0270), T CELL RECEPTOR (0.0310), FC EPSILON RI (0.0380), MTOR (0.0390) |
| SMAD4 | GNRH (0.0000), P53 (0.0020), ERBB (0.0080), NEUROTROPHIN (0.0160), T CELL RECEPTOR (0.0230), MTOR (0.0280), INSULIN (0.0410) |
| STAT1 | T CELL RECEPTOR (0.0020), ERBB (0.0050), P53 (0.0050), CHEMOKINE (0.0060), MTOR (0.0060), GNRH (0.0070), WNT (0.0100), PHOSPHATIDYLINOSITOL SIGNALING SYSTEM (0.0170), MAPK (0.0180), ADIPOCYTOKINE (0.0190), FC EPSILON RI (0.0310) |
| STAT3 | TGF BETA (0.0030), ADIPOCYTOKINE (0.0150), ERBB (0.0300), RIG I LIKE RECEPTOR (0.0300), INSULIN (0.0390) |
| STAT6 | MAPK (0.0000), B CELL RECEPTOR (0.0010), GNRH (0.0010), T CELL RECEPTOR (0.0020), CHEMOKINE (0.0040), FC EPSILON RI (0.0040), TOLL LIKE RECEPTOR (0.0130), RIG I LIKE RECEPTOR (0.0150), NEUROTROPHIN (0.0190), MTOR (0.0220), VEGF (0.0260), PHOSPHATIDYLINOSITOL SIGNALING SYSTEM (0.0340) |
| TCF4 | GNRH (0.0000), ERBB (0.0030), P53 (0.0070), VEGF (0.0120), MAPK (0.0180), FC EPSILON RI (0.0270 |
| TP53 | P53 (0.0340), WNT (0.0420), CHEMOKINE (0.0450), VEGF (0.0460), ERBB (0.0470) |

**Supplementary Table 2** – List of the 40 kinases’ family tested with the GSEA analysis on the ranked list of modulators produced by DMI.

| **SubFamily** | **Kinases (official Gene Symbol)** |
| --- | --- |
| AMPK | BRSK2, SIK1, SIK3 |
| APG1/unc-51/ULK1 | ULK1, ULK2, ULK4 |
| AXL/UFO | AXL, MERTK, TYRO3 |
| Aurora | AURKA, AURKB, AURKC |
| CDC2/CDKX | CDK1, CDK10, CDK12, CDK13, CDK14, CDK16, CDK17, CDK18, CDK19, CDK2, CDK20, CDK4, CDK5, CDK6, CDK9, CDKL1, CDKL2, CDKL3, CDKL5, ICK, MAK |
| CDC5/Polo | PLK1, PLK2, PLK3, PLK4 |
| CSF-1/PDGF receptor | CSF1R, FLT1, FLT3, FLT4, KDR, KIT, PDGFRA, PDGFRB |
| CaMK | CAMK1, CAMK1D, CAMK1G, CAMK2A, CAMK2B, CAMK2G, CAMK4, CASK, DCLK1 |
| Casein kinase I | CSNK1A1, CSNK1D, CSNK1E, CSNK1G1, CSNK1G2, CSNK1G3 |
| DAP kinase | DAPK1, DAPK2, DAPK3, STK17A, STK17B |
| DMPK | CDC42BPA, CDC42BPB, DMPK |
| EGF receptor | EGFR, ERBB2, ERBB3, ERBB4 |
| Ephrin receptor | EPHA1, EPHA2, EPHA3, EPHA4, EPHA5, EPHA7, EPHB1, EPHB2, EPHB3, EPHB4, EPHB6 |
| Fibroblast growth factor receptor | FGFR1, FGFR2, FGFR3, FGFR4 |
| GCN2 | EIF2AK1, EIF2AK2, EIF2AK3 |
| GPRK | ADRBK1, ADRBK2, GRK1, GRK4, GRK5, GRK6 |
| HIPK | HIPK1, HIPK2, HIPK3 |
| I-kappa-B kinase | IKBKB, IKBKE, TBK1 |
| Insulin receptor | ALK, DDR1, DDR2, IGF1R, INSR, INSRR, LTK, NTRK1, NTRK2, NTRK3, PTK7, ROS1 |
| JAK | JAK1, JAK2, JAK3, TYK2 |
| Lammer | CLK1, CLK2, CLK3, CLK4 |
| MAPKKK | MAP3K1, MAP3K10, MAP3K11, MAP3K12, MAP3K13, MAP3K14, MAP3K2, MAP3K3, MAP3K4, MAP3K5, MAP3K6, MAP3K7, MAP3K8, MAP3K9 |
| MAPKK | MAP2K1, MAP2K2, MAP2K3, MAP2K4, MAP2K5, MAP2K6, MAP2K7, PBK |
| MAPK | MAPK1, MAPK10, MAPK11, MAPK12, MAPK13, MAPK14, MAPK3, MAPK4, MAPK6, MAPK7, MAPK8, MAPK9, NLK |
| MARK | MARK1, MARK2, MARK3, MARK4 |
| MNB/DYRK | DYRK1A, DYRK1B, DYRK2, DYRK3, DYRK4 |
| NIMA | NEK1, NEK11, NEK2, NEK3, NEK4, NEK7, NEK9 |
| PKC | PKN1, PKN2, PRKCA, PRKCB, PRKCD, PRKCE, PRKCG, PRKCH, PRKCI, PRKCQ, PRKCZ |
| PKD | PRKD1, PRKD2, PRKD3 |
| Pelle | IRAK1, IRAK3, IRAK4 |
| RAC | AKT1, AKT2, AKT3 |
| RAF | ARAF, BRAF, RAF1 |
| S6 kinase | RPS6KA1, RPS6KA2, RPS6KA3, RPS6KA4, RPS6KA5, RPS6KA6, RPS6KB1, RPS6KB2, RPS6KC1 |
| SNF1 | HUNK, MELK, NUAK1, NUAK2, PRKAA2 |
| SRC | BLK, FGR, FRK, FYN, HCK, LCK, LYN, SRC, YES1 |
| STE20 | MAP4K1, MAP4K2, MAP4K3, MAP4K4, MAP4K5, MINK1, OXSR1, PAK1, PAK2, PAK3, PAK4, PAK6, PAK7, SLK, STK10, STK24, STK25, STK3, STK39, STK4, STRADA, TAOK2, TAOK3, TNIK |
| TEC | BMX, BTK, ITK, TEC, TXK |
| TGFB receptor | ACVR1, ACVR1B, ACVR2A, ACVR2B, ACVRL1, AMHR2, BMPR1A, BMPR1B, BMPR2, TGFBR1, TGFBR2 |
| VRK | VRK1, VRK2, VRK3 |
| cAMP | PRKACA, PRKACB, PRKACG, PRKX |

**Supplementary Table 3** – List of signaling pathways used. For each signaling pathway are reported the involved kinases.

| **Signaling Pathways** | **Involved Kinases (Official Gene Symbol)** |
| --- | --- |
| MAPK | AKT1, AKT3, EGFR, FGFR1, FGFR2, FGFR3, IKBKB, MAP2K1, MAP2K2, MAP2K3, MAP2K6, MAP3K1, MAP3K11, MAP3K14, MAP3K2, MAP3K3, MAP3K4, MAP3K5, MAP3K6, MAP3K7, MAP3K8, MAP4K1, MAP4K2, MAP4K3, MAP4K4, MAPK1, MAPK10, MAPK12, MAPK13, MAPK14, MAPK9, MAPKAPK2, MAPKAPK3, MAPKAPK5, MKNK1, MKNK2, NLK, NTRK2, PAK1, PAK2, PDGFRA, PDGFRB, PRKACB, PRKCA, PRKCB, PRKX, RAF1, RPS6KA1, RPS6KA2, RPS6KA3, RPS6KA5, TAOK3, TGFBR2 |
| ERBB | ABL1, AKT1, AKT3, ARAF, CAMK2B, CAMK2G, CDKN1A, EGFR, ERBB2, ERBB3, GSK3B, MAP2K1, MAP2K2, MAPK1, MAPK10, MAPK9, PAK1, PAK2, PAK3, PAK6, PIK3CA, PRKCA, PRKCB, PTK2, RAF1, RPS6KB1, SHC1 |
| CALCIUM | CAMK2B, CAMK2G, EGFR, ERBB2, ERBB3, ITPKA, MYLK, PDGFRA, PDGFRB, PHKA1, PHKB, PRKACB, PRKCA, PRKCB, PRKX, PTK2B |
| CHEMOKINE | ADRBK1, AKT1, AKT3, CCL2, CCL5, CCL8, CSK, FGR, GRK5, GRK6, GSK3B, HCK, IKBKB, ITK, JAK2, LYN, MAP2K1, MAPK1, PAK1, PIK3CA, PRKACB, PRKCB, PRKCD, PRKCZ, PRKX, PTK2, PTK2B, RAF1, ROCK1, ROCK2, SHC1 |
| PHOSPHATIDYLINOSITOL SIGNALING SYSTEM | ITPKA, PIK3C3, PIK3CA, PRKCA, PRKCB |
| P53 | ATM, ATR, CCNB1, CCND1, CCND3, CDK1, CDK2, CDK4, CDK6, CDKN1A, CHEK1 |
| MTOR | AKT1, AKT3, CAB39, MAPK1, PIK3CA, RPS6KA1, RPS6KA2, RPS6KA3, RPS6KB1, ULK1 |
| WNT | CAMK2B, CAMK2G, CCND1, CCND3, CSNK1A1, CSNK1E, CSNK2A2, CSNK2B, GSK3B, MAP3K7, MAPK10, MAPK9, NLK, PRKACB, PRKCA, PRKCB, PRKX, ROCK1, ROCK2 |
| HEDGEHOG | CSNK1A1, CSNK1E, CSNK1G2, CSNK1G3, GSK3B, PRKACB, PRKX |
| TGF BETA | ACVR1, BMPR1A, BMPR2, LTBP1, MAPK1, ROCK1, ROCK2, RPS6KB1, TGFBR2 |
| VEGF | AKT1, AKT3, KDR, MAP2K1, MAP2K2, MAPK1, MAPK12, MAPK13, MAPK14, MAPKAPK2, MAPKAPK3, PIK3CA, PRKCA, PRKCB, PTK2, RAF1 |
| TOLL LIKE RECEPTOR | AKT1, AKT3, CCL5, IKBKB, IRAK1, MAP2K1, MAP2K2, MAP2K3, MAP2K6, MAP3K7, MAP3K8, MAPK1, MAPK10, MAPK12, MAPK13, MAPK14, MAPK9, PIK3CA, RIPK1, TBK1 |
| NOD LIKE RECEPTOR | CCL2, CCL5, CCL8, IKBKB, MAP3K7, MAPK1, MAPK10, MAPK12, MAPK13, MAPK14, MAPK9 |
| RIG I LIKE RECEPTOR | IKBKB, MAP3K1, MAP3K7, MAPK10, MAPK12, MAPK13, MAPK14, MAPK9, RIPK1, TBK1 |
| JAK STAT | AKT1, AKT3, CCND1, CCND3, JAK1, JAK2, PIK3CA, PIM1, TYK2 |
| T CELL RECEPTOR | AKT1, AKT3, CDK4, FYN, GSK3B, IKBKB, ITK, LCK, MAP2K1, MAP2K2, MAP3K14, MAP3K7, MAP3K8, MAPK1, MAPK12, MAPK13, MAPK14, MAPK9, PAK1, PAK2, PAK3, PAK6, PDK1, PIK3CA, PRKCQ, RAF1, ZAP70 |
| B CELL RECEPTOR | AKT1, AKT3, BTK, GSK3B, IKBKB, LYN, MAP2K1, MAP2K2, MAPK1, PIK3CA, PRKCB, RAF1, SYK |
| FC EPSILON RI | AKT1, AKT3, BTK, FYN, LYN, MAP2K1, MAP2K2, MAP2K3, MAP2K6, MAPK1, MAPK10, MAPK12, MAPK13, MAPK14, MAPK9, PDK1, PIK3CA, PRKCA, PRKCB, PRKCD, RAF1, SYK |
| NEUROTROPHIN | ABL1, AKT1, AKT3, CAMK2B, CAMK2G, CSK, GSK3B, IKBKB, IRAK1, IRAK3, IRS1, MAP2K1, MAP2K2, MAP3K1, MAP3K3, MAP3K5, MAPK1, MAPK10, MAPK12, MAPK13, MAPK14, MAPK9, MAPKAPK2, NTRK2, PDK1, PIK3CA, PRKCD, RAF1, RPS6KA1, RPS6KA2, RPS6KA3, RPS6KA5, SHC1 |
| INSULIN | AKT1, AKT3, ARAF, GSK3B, IKBKB, INSR, IRS1, MAP2K1, MAP2K2, MAPK1, MAPK10, MAPK9, MKNK1, MKNK2, PHKA1, PHKB, PIK3CA, PRKACB, PRKAG2, PRKCI, PRKCZ, PRKX, RAF1, RPS6KB1, SHC1 |
| GNRH | CAMK2B, CAMK2G, EGFR, MAP2K1, MAP2K2, MAP2K3, MAP2K6, MAP3K1, MAP3K2, MAP3K3, MAP3K4, MAPK1, MAPK10, MAPK12, MAPK13, MAPK14, MAPK9, PRKACB, PRKCA, PRKCB, PRKCD, PRKX, PTK2B, RAF1 |
| ADIPOCYTOKINE | AKT1, AKT3, IKBKB, IRS1, JAK2, MAPK10, MAPK9, PRKAG2, PRKCQ |

**Supplementary Table 3 –** the 14 transcription factors tested using the DMI method.

| **Official Symbol** | **Complete Name** |
| --- | --- |
| CDX2 | Caudal Type Homeo Box Transcription Factor 2 |
| E2F1 | Retinoblastoma-Associated Protein 1 |
| ELK1 | ETS domain-containing protein Elk-1 |
| ETS1 | V-ets erythroblastosis virus E26 oncogene homolog 1 (avian) |
| GATA1 | GATA-binding protein 1 |
| GATA2 | GATA-binding protein 2 |
| MYC | Myelocytomatosis oncogene |
| SMAD3 | SMAD family member 3 |
| SMAD4 | SMAD family member 4 |
| STAT1 | Signal transducer and activator of transcription 1 |
| STAT3 | Signal transducer and activator of transcription 3 |
| STAT6 | Signal transducer and activator of transcription 6 |
| TCF4 | Immunoglobulin Transcription Factor 2 |
| TP53 | Tumor protein p53 |

# 4. References

1. Gretton, A., *Consistent Nonparametric Tests of Independence.* J. Mach. Learn. Res., 2010. **99**: p. 1391-1423.

2. Fukumizu, K., et al. *Kernel measures of conditional dependence*. in *In Adv. NIPS*. 2008.

3. Sardiello, M., et al., *A gene network regulating lysosomal biogenesis and function.* Science, 2009. **325**(5939): p. 473-7.
